# The Versatility of Sirtuin-1 in Endocrinology and Immunology

**DOI:** 10.3389/fcell.2020.589016

**Published:** 2020-11-19

**Authors:** Fahmida Rasha, Brianyell McDaniel Mims, Isabel Castro-Piedras, Betsy J. Barnes, Matthew B. Grisham, Rakhshanda Layeequr Rahman, Kevin Pruitt

**Affiliations:** ^1^Department of Immunology and Molecular Microbiology, Texas Tech University Health Sciences Center, Lubbock, TX, United States; ^2^Laboratory of Autoimmune and Cancer Research, Center for Autoimmune Musculoskeletal and Hematopoietic Disease, The Feinstein Institutes for Medical Research, Manhasset, NY, United States; ^3^Department of Molecular Medicine and Department of Pediatrics, Zucker School of Medicine at Hofstra-Northwell, Hempstead, NY, United States; ^4^Department of Surgery, Texas Tech University Health Sciences Center, Lubbock, TX, United States

**Keywords:** SIRT1, SIRTUIN, endocrinology, immunology, autoimmune disease, cancer, development, therapy

## Abstract

Sirtuins belong to the class III family of NAD-dependent histone deacetylases (HDAC) and are involved in diverse physiological processes that range from regulation of metabolism and endocrine function to coordination of immunity and cellular responses to stress. Sirtuin-1 (SIRT1) is the most well-studied family member and has been shown to be critically involved in epigenetics, immunology, and endocrinology. The versatile roles of SIRT1 include regulation of energy sensing metabolic homeostasis, deacetylation of histone and non-histone proteins in numerous tissues, neuro-endocrine regulation via stimulation of hypothalamus-pituitary axes, synthesis and maintenance of reproductive hormones via steroidogenesis, maintenance of innate and adaptive immune system via regulation of T- and B-cell maturation, chronic inflammation and autoimmune diseases. Moreover, SIRT1 is an appealing target in various disease contexts due to the promise of pharmacological and/or natural modulators of SIRT1 activity within the context of endocrine and immune-related disease models. In this review we aim to provide a broad overview on the role of SIRT1 particularly within the context of endocrinology and immunology.

## Introduction

There are four classes of histone deacetylases (HDACs), namely, class I, II, III, and IV. HDAC1 – HDAC11 belong to class I, II, and IV while sirtuins belong to the class III HDAC family. Sirtuin-1 (SIRT1) is the most well-studied member of this family and its activity is NAD+-dependent, unlike the class I, II, and IV HDACs. Early on the yeast counterpart (Sir2) was shown to play a critical role in regulating DNA accessibility. However, over the last decade our understanding of the function and targets of sirtuins has increased. SIRT1 is an evolutionary conserved enzyme whose presence can be traced to archaea. The number of sirtuin family members, varies across species and varies from a single family member in bacteria to seven in mammals (Frye, 2000; Greiss and Gartner, 2009). Notably, human sirtuins are involved in a myriad of cellular processes that impact wide-ranging cellular processes from T cell differentiation to endocrine function. As key regulators of homeostasis, dysregulated sirtuin activity is associated with a wide spectrum of diseases. Even though our understanding of their role in aging, cancer, metabolic syndrome, neuropathologies, and autoimmune diseases (ADs) is increasing, the complexity of their contribution continues to make it difficult to draw firm conclusions about causation or mechanisms. However, regardless of this complexity, there is a consensus in recognizing that sirtuins may serve as potential therapeutic targets. This review will focus on SIRT1 and will discuss its role in the context of epigenetics, immunology, and endocrinology. It will provide a broad historical overview and will also highlight recent discoveries of the importance of post-translational acetylation particularly within the context of endocrinology and immunology. We point the reader to previous excellent reviews that focus more on the role of SIRT1 in aging (Longo and Kennedy, 2006), adaptive cellular responses (Anastasiou and Krek, 2006), and endocrine signaling (Yang et al., 2006).

### Overview of Histone Deacetylases

Histone acetyltransferases (HATs), also referred to as lysine acetyltransferases (KATs), were the first enzymes shown to modify histones (Allfrey et al., 1964; Marmorstein, 2001; Allis et al., 2007; Pruitt, 2016) and have since been shown to modify numerous non-histone proteins (Saxena et al., 2013; Knyphausen et al., 2015; Pandey et al., 2015; Di et al., 2016; Tapias and Wang, 2017; Molehin et al., 2018). Protein acetylation involves acetyl-coenzyme A (acetyl-CoA) and therefore will be influenced by the cellular metabolic state (Peleg et al., 2016). While HATs/KATs are the writers of acetylation, the erasers are categorized as class I, II, III, and IV histone deacetylases. Additionally, the term lysine deacetylase (KDAC) will sometimes be used interchangeably with HDAC because this family deacetylates numerous non-histone proteins. The mechanism of deacetylation differentiates class I/II/IV HDACs from the sirtuins (class III). More than three decades ago histone deacetylase activity could be demonstrated; however, it was not until 1996 that the first HDAC was identified (Taunton et al., 1996). Subsequently, multiple HDAC family members were cloned and characterized (Ng and Bird, 2000). There are currently at least 18 distinct members of HDAC family. Class I HDACs (HDAC1, 2, 3, and 8) are largely nuclear proteins and are homologous to yeast RPD3 protein (Emiliani et al., 1998; Yang and Seto, 2008). Class II HDACs (HDAC4, 5, 6, 7, 9, and 10) shuttle between the nucleus and cytoplasm and are homologous to yeast Hda1 protein and are generally expressed in a tissue-specific manner (Fischle et al., 1999; Grozinger et al., 1999). HDAC 11 does not conform to the sequence similarity of class I or II, so it is considered a class IV HDAC (Gregoretti et al., 2004). In mammals, the sirtuin family (SIRT1-7) comprises the class III HDACs, and among them SIRT1 is the human homolog of yeast Sir2 (Blander and Guarente, 2004). While the class I, II, and IV, deacetylases are Zn^2+^-dependent deacetylases, sirtuins catalyze the deacetylation reaction in a NAD^+^-dependent manner. For the removal of every acetyl group from the substrate, one NAD^+^ is hydrolyzed resulting in a deacetylated protein and the reaction products, *O*-acetyl-ADP-ribose and nicotinamide. Nicotinamide is the amide derivative of vitamin B3 and can act as part of a negative feedback inhibitor of sirtuins. The role of the other reaction product, *O*-acetyl-ADP-ribose, is less clear, but some reports suggest that it influences cellular metabolic pathways (Pazienza et al., 2014), chromatin structure (Tung et al., 2017), and epigenetic gene silencing (Wang S. H. et al., 2019).

### Role of SIRT1 in Histone Protein Deacetylation

It is remarkable that billions of cells with identical genomes show tissue-specificity. This is achieved by regulating transcription via a mechanism that links transcription potential with specific chromatin structures and epigenetic states. Chromatin can be defined as the sum of DNA, histones and associated RNA and it not only regulates transcription but also serves to compact and protect DNA. The general chromatin structure is influenced by epigenetic states. In general, “epigenetics” refers to a heritable pattern of gene expression that is not the result of alterations in the primary nucleotide sequence of a gene. The specific epigenetic state is the result of dynamic and reversible covalent modifications to DNA or post-translational modifications to histones. These so called “epigenetic marks” involve post-translational modifications to histone tails (such as acetylation, phosphorylation, ubiquitylation, methylation, and sumoylation) and methylation of DNA (Martin and Zhang, 2005; Nightingale et al., 2006; Ting et al., 2006). SIRT1 plays a role in deacetylating key lysines on histone H1, H3, and H4 which will directly impact chromatin structure and the accessibility of promoters to transcriptional activators (Pruitt et al., 2006; Alexander et al., 2007; Dawson and Kouzarides, 2012; Pruitt, 2016). SIRT1 has been shown to deacetylate a number of critical lysines such as histone H3 lysine 9 (H3K9) and histone H4 lysine 16 (H4K16) which collectively determine whether a gene is transcribed. CpG islands are typically associated with the promoter regions of genes where the methylation status correlates with transcription (Herman and Baylin, 2003). CpG methylation often works in concert with other epigenetic modifications such as histone hypoacetylation. The extent of histone acetylation impacts chromatin structure and gene transcription and the acetylation status of H3K9 and H4K16 influences recruitment of co-repressors and gene expression (Bannister et al., 2001; Nakayama et al., 2001). SIRT1 influences the epigenome in two distinct ways. First, as discussed above, it can directly deacetylate a number of key lysines on multiple histones. SIRT1 has been shown to regulate H3K9 and H4K16 at promoters wherein DNA methylation is enriched and contribute to epigenetic silencing of genes that modulate Wnt signaling (Pruitt et al., 2006). Second, SIRT1 can deacetylate other epigenetic enzymes that add (writers) or remove (erasers) epigenetic marks. In the case of an epigenetic writer that deposits DNA methylation on cytosine residues, DNA methyl transferase 1 (DNMT1) is a target of SIRT1 mediated deacetylation. However, the effect of DNMT1 deacetylation depends on the location of the lysine targeted and will dictate whether its DNA methyltransferase-dependent activity is augmented or whether its methyltransferase-independent transcription repression is limited (Peng et al., 2011). Regardless of whether SIRT1 is deacetylating histones or epigenetic writers, readers or erasers, the net effect will lead to important epigenomic changes. One particularly interesting example involves SIRT1 regulating the onset of puberty based on metabolic and nutritional cues. Kiss1 is a gene that controls the onset of female puberty based on its expression which is controlled by epigenetic mechanisms. Interestingly, SIRT1 is expressed in hypothalamic neurons and suppresses Kiss1 expression by partnering with polycomb group (PcG) proteins that mediate epigenetic silencing. With the onset of puberty, SIRT1 is evicted from the Kiss1 promoter, the gene is activated, and the brake on the hypothalamic-pituitary-gonad axis is released. Undernutrition has been shown to repress the onset of puberty whereas obesity has been shown to induce early onset (Vazquez et al., 2018). Moreover, beyond the effects in neurons, SIRT1 signaling in astrocytes also contributes to metabolic and reproductive regulation and deacetylase-deficient SIRT1 impairs the estrous cycles, decreases luteinizing hormone (LH) surges, and leads to fewer corpora lutea (Choi et al., 2019). While SIRT1 influences the epigenetic activation and repression of many critical genes, the recurring mechanism of its involvement will be to either directly regulate chromatin structure via histone-deacetylation or indirectly regulate it via deacetylation of chromatin modifying enzymes.

### Role of SIRT1 in Non-histone Protein Deacetylation

#### Acetylation as a Regulator of Protein Function

Introduction of acetyl functional groups to histones was discovered more than five decades ago when it was associated with more accessible DNA (Allfrey et al., 1964). However, in recent years the study of acetylation is expanding outside its well-known histone context. Using high-resolution mass spectroscopy, Choudhary and his team were able to make an acetylome portrait by identifying 3600 lysine acetylation sites on 1750 proteins (Choudhary et al., 2009). Acetylation is recognized as one of the most common epigenetic modifications (Polevoda and Sherman, 2000) while also shaping the activity, location and stability of non-histone proteins. Acetylation regulates pathways in major cellular compartments including nucleus, plasma membrane, cytosol, and mitochondria and regulates metabolism, cell growth and insulin/insulin-like signaling (Pirola et al., 2012; Shi and Tu, 2014). Lysine acetylation of mitochondrial proteins was underappreciated until recently even though more than 20% of mitochondrial proteins are acetylated (Kim S. C. et al., 2006). Ghanta et al. (2013) propose that the mitochondrial acetylome acts as an energy consumption/storage switch. For instance, when a cell is exposed to constant energy excess or over-nutrition, malate dehydrogenase 2 (MDH2) is activated by hyperacetylation and inactivates energy consumption and promotes energy storage (e.g., gluconeogenesis) (Zhao et al., 2010). However, many other mitochondrial enzymes including superoxide dismutase 2 (Qiu et al., 2010), acetyl-CoA synthetases (Hallows et al., 2006), and glutamate dehydrogenase (Schlicker et al., 2008) are regulated by acetylation. Disturbance in lysine acetyl transferase activity causes cells to lose their ability to appropriately interpret the stress cues. Because of the reversibility of acetylation/deacetylation enzymatic reactions, there is increasing interest in exploiting this post-translational modification for therapeutic purposes. Currently, class I/II histone deacetylases are the targets of several molecule inhibitors in various phases of clinical trials, where some agents have yielded highly promising results (Johnstone, 2002; Rosato and Grant, 2004).

#### SIRT1 Deacetylates Many Non-histone Proteins

One of the challenges of sorting out the impact of pharmacologic inhibition of histone modifying enzymes is that non-histone proteins are also substrates. For example, SIRT1 knockdown in multiple colon cancer and breast cancer cells was shown to induce p53 activation by acetylation in the absence of conventional stress and induced either apoptosis or growth arrest, depending on the cell type (Ford et al., 2005). In a different study, SIRT1 was shown to bind and deacetylate estrogen related receptor α (ERRα) which resulted in enhancement of its DNA-binding potential (Wilson et al., 2010). Interestingly, ERRα is one of the transcription factors that regulates transcription of the CYP19A1 gene which encodes for the aromatase protein. Aromatase is the enzyme that converts testosterone to estrogen in the estrogen biosynthesis pathway and aromatase inhibitors are used for treatment of breast cancer. Another report demonstrated that inhibition of SIRT1 reduced aromatase mRNA and protein levels in estrogen receptor negative (ER−) breast cancer cells possibly due to loss of ERRα binding to the aromatase promoter and subsequent inhibition of aromatase transcription (Holloway et al., 2013). While SIRT1 is shown to promote many cancer phenotypes, a positive regulator of many cancer hallmarks (Hanahan and Weinberg, 2011), and is overexpressed in human cancer specimens compared to normal tissue, some reports from transgenic mouse models have identified tumor suppressor properties of SIRT1 (Firestein et al., 2008). Some of the controversy regarding whether SIRT1 acts in an oncogenic or tumor suppressive manner may be better reconciled as we consider the difference in multiple experimental designs. For example, two studies came to different conclusions regarding the *in vivo* influence of SIRT1 on tumor biology. Both studies used APC^min/+^ mouse models (with germline mutation in the APC tumor suppressor gene) that enabled spontaneous adenomatous polyps and hyperplasia resulting in colon cancer. Both studies investigated the role of SIRT1 in colon tumorigenesis. Firestein et al. (2008) used conditional overexpression of SIRT1 in the intestine to show fewer polyps. However, Leko et al. (2013) used a conditional enterocyte-specific SIRT1 knockout and observed reduced tumor size and number of polyps, with no changes in proliferation but an increase in apoptosis of tumor cells in knockouts vs. wild type APC^min/+^mice. Since the gain-of-function and loss-of function phenotypes were similar, Leko et al. (2013) argued that super-physiological levels of SIRT1 (as in the Firestein study) due to overexpression, might somehow be causing stoichiometry changes in protein complexes resulting in the inactivation of the overexpressed protein. These types of differences in experimental design need to be weighed as studies seeking to label chromatin regulators as tumor suppressers or oncogenes. More recent studies have identified several novel non-histone targets of SIRT1 including DNA methylation readers (MeCP2) and Dishevelled proteins which are critical regulators of Wnt signaling (Saxena et al., 2013; Knyphausen et al., 2015; Pandey et al., 2015; Di et al., 2016; Tapias and Wang, 2017; Molehin et al., 2018). Both MeCP2 and DVL1 and 3 are deacetylated by SIRT1. MeCP2 deacetylation regulates its binding to co-repressors (Saxena et al., 2013; Knyphausen et al., 2015; Pandey et al., 2015; Di et al., 2016; Tapias and Wang, 2017; Molehin et al., 2018). SIRT1 activity regulates DVL1 binding to TIAM1, an activator of Rac that is important for cell migration. SIRT1-mediated DVL1 deacetylation on critical sites in the DIX and PDX domain regulates its sub-cellular localization and ability to activate CYP19A1 promoters (Sharma et al., 2018, 2019).

## SIRT1 and Endocrine Function

The importance of SIRT1 in metabolism has been clearly established since its discovery and for a more in-depth review of the role of sirtuins in metabolism, we point the reader to other excellent reviews discussing sirtuins role in lipid metabolism (Simmons et al., 2015; Ye et al., 2017). In the following sections we will focus on the role of SIRT1 in the regulation of endocrine signaling and hormone production.

### Connections With Hypothalamus-Pituitary Axes

Recent publications clearly demonstrate SIRT1 involvement in transcription and metabolic processes via hormonal production and homeostasis regulation in the neuroendocrine system (Yang et al., 2006; Cohen et al., 2009; Chang and Guarente, 2014). The hypothalamic pituitary axes play an integral role in mediating SIRT1 specific hormonal control in the neuroendocrine system. The hypothalamus regulates body temperature, hunger, thirst, energy expenses, emotion/behavior, circadian rhythm of organisms and thus maintains body homeostasis. In addition, hypothalamic cues such as synthesis or secretion of hypothalamic hormones induce or inhibit synthesis or secretion of pituitary hormones (Markakis, 2002; Xie and Dorsky, 2017). Pituitary hormones regulate hormone secretion by respective target organs via systemic blood circulation and hence a pathway is established from hypothalamus to target organs via the pituitary glands known as the ‘hypothalamus-pituitary axis.’ There are four major hypothalamus-pituitary axes found in the circulation based on specific target organ. These are: (i) hypothalamus-pituitary-adrenal (HPA) axis, (ii) hypothalamus-pituitary-thyroid (HPT) axis, (iii) hypothalamus-pituitary-gonadal (HPG) axis, and (iv) somatotropic axis (Yamamoto and Takahashi, 2018). Below we will briefly discuss the connection between SIRT1, hypothalamus and four different hypothalamus-pituitary axes in regulation of endocrine synthesis and signaling.

#### Hypothalamic SIRT1’s Role in Maintenance of Body Homeostasis

Researchers reported SIRT1 expression in steroidogenic factor 1 (SF1), proopiomelanocortin (POMC) and agouti related peptide (AgRP) neurons of the ventromedial hypothalamic nucleus (VMH) and arcuate nucleus (ARH) respectively (Sasaki et al., 2014; Orozco-Solis et al., 2015). In addition, SF1 specific SIRT1 deletion induced insulin resistance while SIRT1 overexpression in SF1 resulted in insulin sensitivity and prevention of diet-induced obesity in skeletal muscles of transgenic type 2 diabetic mice (Ramadori et al., 2011). Moreover, targeted SIRT1 overexpression in POMC and/or AgRP neurons prevented weight gain and improved energy expenditure *in vivo* (Sasaki et al., 2014). Hypothalamic SIRT1 is also involved in regulation of feeding behavior, emotion, and physiological rhythms. For instance, SIRT1 regulates *in vitro* transcription of anorexigenic/orexigenic neuropeptides in HEK293 cells via Foxo1-induced AgRP promoter activity (Sasaki et al., 2010) while SIRT1 upregulation in the brain induced *in vivo* anxiety via increased transcription of genes encoding monoamine oxidase-A (Mo-A) enzyme (Mo-A inhibitors are involved in clinical depression and anxiety treatment) (Libert et al., 2011). Additionally, significant increase in food anticipatory activity was found among transgenic mice with brain-specific SIRT1 overexpression while SIRT1 knockdown reversed such activity in mice (Satoh et al., 2010). These results indicate crucial region-centric role of hypothalamic SIRT1 and its possible involvement in modulating hypothalamus-pituitary axes’ metabolic functions in the neuroendocrine system.

The HPA axis is an important part of the neuroendocrine system that controls survival, metabolism, immunity, appetite, stress, emotion, and behavior of an organism. Stress response begins in the HPA axis with corticotrophin releasing hormone (CRH) secretion by the hypothalamic paraventricular nucleus (PVN) and via POMC production by the pituitary corticotroph. POMC respectively cleaved by prohormone convertase 1 and 2 (PC1 and PC2) in the pituitary corticotroph, thus results in synthesis of adrenocorticotropic hormone (ACTH) and α-melanocyte stimulating hormone (α-MSH). ACTH then triggers glucocorticoid production by the adrenal cortex responding to stress conditions (Yates et al., 2013; Yamamoto and Takahashi, 2018). Though researchers reported lack of connection between POMC specific SIRT1 induction/ablation with HPA axis activation during *in vivo* fasting conditions (Ramadori et al., 2010), SIRT1 increased PC2 levels in the PVN resulting in increased CRH production and HPA axis stimulation in high-fat diet fed mice (Toorie et al., 2016). Moreover, SIRT1 activator, resveratrol increased PC1 and PC2 levels while SIRT1 inhibitor, EX-527, decreased PC1 and PC2 levels in mouse corticotroph cell line (AtT20) suggesting indirect HPA axis modulating roles of SIRT1 via PC1 and PC2 (Toorie and Nillni, 2014; Toorie et al., 2016). Further, SIRT1 overexpression reduced corticosterone (CORT) mediated autophagy and induced apoptosis while SIRT1 knockdown reversed the results during chronic cellular stress *in vitro* indicating SIRT1 involvement in stress management via HPA axis (Jiang et al., 2019). Hence, these results clearly denote SIRT1 levels in hypothalamus, pituitary and adrenal glands regulate HPA axis functions in response to feeding/fasting and stress conditions.

Next, another major axis in hypothalamus and pituitary connection is the HPT axis which is activated when the hypothalamus senses reduced levels of thyroid hormone in the circulation. This is followed by release of thyrotropin-releasing hormone (TRH) from hypothalamus stimulating thyroid stimulating hormone (TSH) secretion by the pituitary and production of thyroid hormones (TH) by the thyroid organs. Thus normal TH level in the circulation is maintained (Sam and Frohman, 2008). SIRT1 is expressed in TSH producing thyrotroph cells and increases cellular endocytosis via deacetylation of enzyme involved in phosphatidylinositol-4,5-bisphosphate synthesis *in vitro.* On the other hand, *in vivo* SIRT1 knockout reduced TSH secretion via phosphatidylinositol-4-phosphate 5-kinase type 1γ (enzyme) acetylation indicating SIRT1 role in regulating hormone production possibly via HPT axis stimulation (Akieda-Asai et al., 2010). Similarly, serum thyroxin (T4) levels dropped in calorie-restricted SIRT1 knockout (KO) mice followed by reduced physical activity while non-calorie restricted SIRT1-KO mice were hypermetabolic, had inefficient hepatic mitochondria and increased lipid oxidation rates (Boily et al., 2008). In lieu, thyroxin treatment repressed hepatic SIRT1 expression and activity in 48 h fasted mice via TH receptor β indicating negative correlation between serum T4 and hepatic SIRT1 levels *in vivo* (Cordeiro et al., 2013). These data represent close connection between SIRT1 and HPT axis in hormonal regulation and energy metabolism.

The HPG axis plays major role in hypothalamus and pituitary connection via management of reproduction and life cycle. Gonadotropin-releasing hormone (GnRH) secreted from hypothalamic GnRH neurons forms a network which stimulates secretion of LH and follicle-stimulating hormone (FSH) by the pituitary gonadotroph cells followed by production of estrogen or testosterone by the gonads (Sam and Frohman, 2008). SIRT1 knockout mice showed diminished HPG endocrine signaling as identified by twofold reduction in hypothalamic GnRH expression, followed by reduced serum LH and FSH levels and arrested spermatogenesis as confirmed by histological analyses of animal testes (Kolthur-Seetharam et al., 2009). In addition, *in vitro* GnRH administration reduced post-transcriptional SIRT1 levels via induced action of miR-132/212 in the pituitary and downregulation of SIRT1 dependent Foxo1 deacetylation (Lannes et al., 2015). Besides recent investigations are also focused on SIRT1 role in puberty and infertility associated diseases during energy deficiency and abundance (Tatone et al., 2018; Vazquez et al., 2019), but their connections with HPG axis are yet to be explored.

In the somatotropic axis, the hypothalamus induces growth hormone (GH) secretion by pituitary somatotroph cells via releasing GH releasing hormone (GHRH). GH then binds to hepatic GH receptors leading to increased insulin like growth factor-1 (IGF-1) levels in the circulation. Additionally, local IGF-1 production by various tissues like bone, muscle and adipose tissue is also stimulated by GH (Sam and Frohman, 2008). Studies across different species reported progressive interaction between SIRT1 and the somatotropic pathways (Tissenbaum and Guarente, 2001; Al-Regaiey et al., 2005; Gan et al., 2005; Parrella and Longo, 2010) where reduced functionality in these pathways results in lifespan extension in both invertebrate and mammalian models (Bartke et al., 2013; Milman et al., 2016). SIRT1 brain-specific knockout (BS-KO) mice demonstrated dwarfism, smaller pituitary gland and diminished circulatory and hepatic levels of GH and IGF-1 though serum levels of other pituitary hormones remained unaltered in those animals (Cohen et al., 2009). This might be due to hypothalamic dysfunctions in absence of SIRT1 in those animals (Cohen et al., 2009), but how hypothalamic SIRT1 regulates somatotropic axis in hypothalamus-pituitary crosstalk is not known yet. Similarly, SIRT6 BS-KO mice exhibited postnatal growth retardation along with reduced serum GH and IGF-1 levels, however, hypothalamic GHRH and somatotropin release inhibiting factor (SRIF) levels were unchanged (Schwer et al., 2010). Such abnormalities might be due to impaired hypothalamic functions and dysregulated feedback mechanisms in absence of SIRT6 in those animals. Additionally, SIRT1 activation via resveratrol in rat somatotropic cells reduced GH secretory levels both *in vivo* and *in vitro*. Here, SIRT1 activation and overexpression suppressed cAMP response element binding protein (CREB) phosphorylation and glycogen synthase kinase 3 beta (GSK3β) deacetylation via protein phosphatase 1 which, in turn, transcriptionally deactivates CREB resulting in GHRH-mediated GH suppression (Monteserin-Garcia et al., 2013). From the above discussion, it is clear that SIRT1 is involved in regulating hormonal production and functions in the neuroendocrine system via modulating four major hypothalamic-pituitary axes. However, it is critical to understand that sirtuins can switch roles to adapt during low vs. high energy states by modulating hypothalamic-pituitary axes at various steps.

### Connections to Steroidogenesis

Steroidogenesis involves conversion of cholesterol to sex hormones such as androgens, estrogens, and progesterone as well as to other steroid hormones such as glucocorticoids, mineralocorticoids to regulate cellular development and physiology via modulation of transcription and post-transcriptional modifications of various tissue-specific steroidogenic enzymes and co-factors (Bremer and Miller, 2014). SIRT1 can deacetylate histone and non-histone proteins in an energy-sensitive manner and regulate steroid hormone receptor activity. For example, SIRT1 can repress transcription of estrogen and androgen receptors via deacetylation while modulate progesterone receptors transcription via nucleo-cytosolic shuttling of the receptor and induction of slow vs. rapid progesterone response genes (Moore et al., 2012). Particularly, SIRT1 controls the activity of multiple steroid hormones via different mechanisms in cancer. Since SIRT1 overexpression is associated with poor survival and prognostic outcomes including higher lymph node metastases in cancer patients (Wu et al., 2012), post-translational modifications of SIRT1 such as deacetylation, phosphorylation, sumoylation determine hormonal dependence and -independence of multiple cancers through understanding the interactive association between SIRT1 and steroid hormone receptors. Moore et al. (2012) elaborately discussed different mechanisms involved in regulation of SIRT1 and steroid hormone receptors interaction. Below we will briefly discuss steroid hormone receptor modulatory roles of SIRT1 with respect to different diseased conditions.

(i) Regulation of androgen receptors (AR): SIRT1 is able to modulate AR activity via direct deacetylation, leading to inhibition of AR activation, translocation and transcription of AR-dependent genes, for instance in prostate cancer. SIRT1 inhibited AR-dependent prostate cancer cell growth and induced endogenous *AR* target genes repression while SIRT1 antagonists induced expression of AR-responsive target genes (*AR*) both *in vivo* and *in vitro* (Fu et al., 2006; Kojima et al., 2008). Similarly, shRNA mediated SIRT1 knockdown enhanced cell proliferation and reduced autophagy by inhibited phosphorylation of S6K and 4E-BP1 while SIRT1 activation by resveratrol reversed these effects in androgen-responsive prostate cancer cells (Fu et al., 2006; Li et al., 2013). On the other hand, androgen antagonists promote physical association of SIRT1 with transcriptional co-repressor, NCoR and AR, thus recruiting SIRT1 and NCoR to the AR target genes’ promoter region followed by inducing local deacetylation of histone H3K9 in the promoter regions. This finding by Dai et al. (2007) demonstrated ligand-dependent recruitment of SIRT1 into a transcriptional co-repressor complex inducing AR-mediated transcriptional repression for the first time. Apart from this, researchers also reported AR-sensitive prostate cancer progression via (a) transcriptional association between NF-κB and SIRT1 (Jaganathan et al., 2014), (b) SIRT1 dependent transcriptional upregulation of VEGF-C and downregulation of the IGF-1R pathway (Li et al., 2005), and (c) via endogenous SIRT1 mediated induction of autophagy in cultured cells (Powell et al., 2011). Meanwhile, SIRT1 activation in BRCA1 overexpressed breast cancer cells inhibited AR expression and AR-stimulated cancer cell proliferation (Zhang et al., 2016). These results suggest critical role of SIRT1 in regulating cancer-specific AR signaling while loss of SIRT1 activity might result in loss of AR transcriptional control leading to ineffectual hormone-replacement therapy in multiple cancers.

(ii) Regulation of estrogen receptors (ER): SIRT1 leads to repressed ERα translocation from cytosol to nucleus resulting in reduced ERα binding and target gene transcription. SIRT1 expression is highly elevated by stimulated 17-β-estradiol (E2) levels in most ERα-positive breast cancer samples (Elangovan et al., 2011; Lee et al., 2011) while SIRT1 inhibition leads to ER signaling inhibition (Yao et al., 2010) implying SIRT1 role as ERα co-activator. SIRT1 dependent ERα inhibition resulted in E2-mediated suppression of cell growth in both healthy and malignant mammary epithelial cells (Yao et al., 2010). Moreover, SIRT1 has been found to interact with p300, PPARγ, PGC1α, and ERα transcriptomic co-activators thus regulating developmental processes such as chromatin remodeling (Picard et al., 2004; Rodgers et al., 2005). In addition, published work from our lab demonstrated that SIRT1 regulates estrogen production at two levels. First, SIRT1 binds multiple tissue-specific aromatase promoters and its inhibition decreases aromatase transcripts (Holloway et al., 2013; Molehin et al., 2018). Second, the aromatase protein itself is a target of SIRT1-mediated deacetylation (Holloway et al., 2013; Molehin et al., 2018). E2-mediated ERα activation also resulted in p300 mediated stabilization of the receptor via acetylation which can be reversed by SIRT1 (Kim M. Y. et al., 2006) implying SIRT1 inhibitory action on ER are mostly estrogen dependent. However, researchers also reported contradictory SIRT1 role as ERα repressor especially in breast cancer. Resveratrol inhibited breast cancer cell growth and upregulated SIRT1 mRNA and protein expression (Lin et al., 2010) while SIRT1 pharmacological inhibitor, sirtinol and splitomicin along with anti-SIRT1 siRNA demonstrated repressed SIRT1 activity, resulting in inhibited ERα-mediated cancer gene transcription in an estrogen-independent manner. Such SIRT1 inhibition (pharmacological/siRNA) leads to PI3K/Akt dependent ERα inhibition and repressed estrogen-dependent breast cancer cell growth (Moore et al., 2012). In addition, aberrant SIRT1 activity also leads to ER-dependent alterations in FOXO-family transcriptional deregulations (FOXOM1, FOXO3a) especially in triple negative breast cancer (Lee et al., 2016) while SIRT1 overexpression is observed in ER−/Her2+ breast cancer cells with E2 interactions with G-protein coupled ERs (GPER) via activation of EGFR/ERK/c-fos/AP-1 signal transduction pathways (Santolla et al., 2015). Intriguingly, induced cell proliferation via such interaction can be reversed by either SIRT1 inhibition or GPER silencing (Santolla et al., 2015). However, knowledge is still limited regarding the interplay between SIRT1 and GPER modulation in cancer progression. On a different note, SIRT1/ERα signaling has anti-oxidant function in different preclinical models of chronic disease and is involved in reducing oxidative stress, neuroinflammation and preventing arterial stiffness via ameliorating JNK/NF-κB signaling, and via upregulating endothelial nitric oxide synthase (eNOS) expression and reduced eNOS uncoupling, respectively (Khan et al., 2019; Li et al., 2019). Taken together, SIRT1 is integral in regulating ER-mediated transcriptional activity in different disease conditions, especially in cancers.

(iii) Inhibition of progesterone receptors (PR): SIRT1 via nucleo-cytoplasmic translocation of PR, leads to downstream activation of PR-regulated genes. The acetylated PR regulates the nuclear-cytosolic shuttling of the receptor and transcriptional activation of slow vs. rapid PR-responsive genes. For instance, acetylation-mimetics at the PR hinge region show delayed nuclear entry upon progesterone binding while acetylation-deficient mutants induced rapid *c-MYC* (a rapid PR-responsive gene) gene expression compared to wild type (Daniel et al., 2010). Additionally, SIRT1/c-MYC builds a negative feedback loop in such a way that c-MYC binds to SIRT1 promoter region upregulating its expression whereas SIRT1 deacetylates c-MYC reducing its transformative stability (Yuan et al., 2009). This further suppress cell proliferation and induce cell cycle arrest at G1/S phase suggesting PR-dependent SIRT1/c-MYC role in tumorigenesis (Mao et al., 2011). However, it is still not known whether SIRT1 is able to deacetylate PR itself in healthy vs. diseased condition. *In vitro* administration of SIRT1 agonists are also involved in induced progesterone secretion. Resveratrol enhanced serum progesterone levels by threefold after 48 h of culture while visfatin (an adipocytokine and SIRT1 inducer) treatment induced ∼2-fold increase in progesterone secretion compared to basal state in rat and human granulosa cells, respectively (Morita et al., 2012; Reverchon et al., 2013). On the other hand, when T47D breast cancer cells were treated with nicotinamide (NAM), a SIRT1 inhibitor, PR-responsive gene transcription was decreased in a dose-dependent manner. Surprisingly, specific siRNA-mediated SIRT1 inhibition demonstrated SIRT1 independent PR-transcriptional repression. Hence, the authors reported a SIRT1 independent mechanism of NAM which inhibits the coordination of basal transcription machinery assembly of the progesterone-responsive promoter after chromatin remodeling and likelihood of off-target NAM effects apart from SIRT1 inhibitory effects (Aoyagi and Archer, 2008). Therefore, selecting a more specific SIRT1 inhibitor to investigate its role in PR regulation is warranted.

(iv) Regulation of glucocorticoid receptor (GR) signaling: SIRT1 and GR activity can be better described in light of myocyte metabolism. SIRT1 regulates deacetylation of the GR and its ability to interact with co-activator p300 through maintaining cellular NAD/NADH ratio. Induced GR ligand binding activates SIRT1 co-regulatory protein p300 leading to increased uncoupling protein-3 (UCP3) gene transcription (involved in mitochondrial stress response). SIRT1 then represses UCP3 transcription by preventing GR and p300 interaction at the promoter (Amat et al., 2007). Therefore, SIRT1 and GR interaction controls the metabolic state of cells, acting as an energy sensor via cellular NAD/NADH regulation and linking the transcriptional parameters of metabolic genes to the stress response. SIRT1 is involved in preventing glucocorticoid induced muscle wasting and mitochondrial dysfunction as reported by many researchers (Knobloch et al., 2014; Poulsen et al., 2014; Chiu et al., 2018; Ma et al., 2019). Resveratrol prevented dexamethasone-induced acetylation of FOXO1, expression of atrogin-1 and muscle ring finger protein-1 (MuRF1), and protein degradation in cultured myotubes in a SIRT1 dependent manner (Alamdari et al., 2012). Moreover, SIRT1-mediated enhancement of GR transcriptional activity independent of its deacetylation function was reported while SIRT1 knockdown reversed this phenomenon. At a molecular level, SIRT1 can physically interact with GR and glucocorticoid inducible proteins. SIRT1 further co-localizes with GR in the nucleus upon dexamethasone treatment and regulates GR transcriptional activity by cooperating with PGC1α in a deacetylase independent manner as shown in three different human cancer (cervical, colon, hepatic) cell lines (Suzuki et al., 2018). This suggests an important role of SIRT1 and GR interplay in various human diseases.

(v) Regulation of mineralocorticoid receptors (MR): SIRT1 regulates MR via enhanced binding to disruptor of telomeric silencing-1 (DOT1) and thus repression of MR-regulated genes, which is independent of SIRT1 deacetylase activity. The MR and its ligand, aldosterone, maintain electrolyte balance in the kidney via induced renal tubular sodium absorption and increased transcription of epithelial sodium channel α-subunit (α-ENaC) (Fan et al., 2011). SIRT1 modulates aldosterone signaling in a MR-dependent manner via its physical interaction with DOT1, which is a histone methyltransferase. This results in H3K79 global hypermethylation in chromatin and α-ENaC 5’-flanking region leading to transcriptional repression. Although this process requires SIRT1 to support DOT1 hypermethylation, it is independent of SIRT1 deacetylase activity indicating SIRT1 is a novel modulator of aldosterone pathway (Zhang D. et al., 2009). In lieu, SIRT1 overexpression prevented aldosterone-induced mitochondrial damage and podocyte injury via upregulating PGC-1α transcription and translation while resveratrol lessened aldosterone-induced mitochondrial malfunction *in vitro* and in aldosterone-infused mice (Yuan et al., 2012). Additionally, MR regulates obesity-associated cardiovascular complications through regulating adipose tissue mitochondrial functions. Hence, MR antagonists prevented adipocytes senescence *in vivo* via reducing SIRT1 levels and blocking p53/p21 pathway (Lefranc et al., 2019) indicating the possibility of MR and SIRT1 mediated stress response in obesity. Furthermore, SIRT1 regulates adipocyte renin-angiotensin-aldosterone system (RAAS) via targeting MR. When mouse preadipocytes were treated with angiotensin (Ang) II, they showed upregulated MR and reduced SIRT1 levels which was reversed by antioxidant heme oxygenase-1 (HO-1) administration. However, SIRT1 knockdown in preadipocytes by specific siRNA increased lipid accumulation and fatty acid synthase levels *in vitro* indicating SIRT1 mediated beneficial effects of HO-1 in rescuing Ang II-induced impaired MR upregulation in adipose tissue (Lakhani et al., 2019). Together, these data suggest novel MR regulatory roles of SIRT1 both dependent and independent of its deacetylase function in multiple disease conditions.

## Role of SIRT1 in Innate and Adaptive Immunity

**FIGURE 1:**
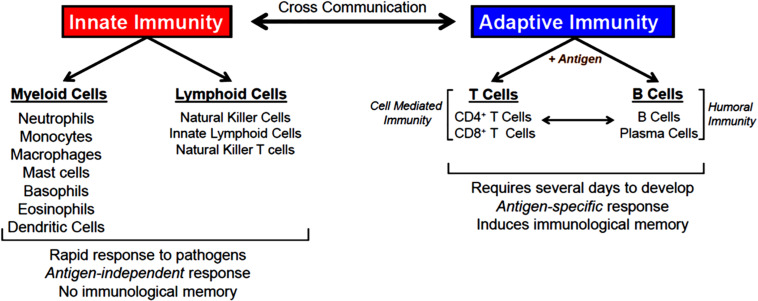
Innate and adaptive immunity. Defense against invading microbes requires well-regulated interactions between innate and adaptive immune cells. Innate immune cells provide rapid responses (hours-days) to pathogens via the action of several different myeloid and lymphoid cells. Innate immune responses are antigen-independent and do not result in immunological memory. Adaptive immune cells (T and B cells) recognize a spectrum of microbial antigens that induce the activation and clonal expansion of thousands of antigen-specific lymphocytes. Highly specialized T and B cell responses to microbial antigens develop over the course of several days to weeks providing the host with long-lived memory T and B cells. T cells are required for cell-mediated immunity, a process by which activated T cells help kill pathogens that have been phagocytosed by macrophages and neutrophils. B cells and their tissue-associate counterparts (plasma cells) are required for humoral immunity via their ability to produce antigen specific antibodies that bind to and eliminate extracellular microorganisms. There are a number of interactions between and within innate and adaptive immune responses that enhance overall protection from pathogens.

**FIGURE 2:**
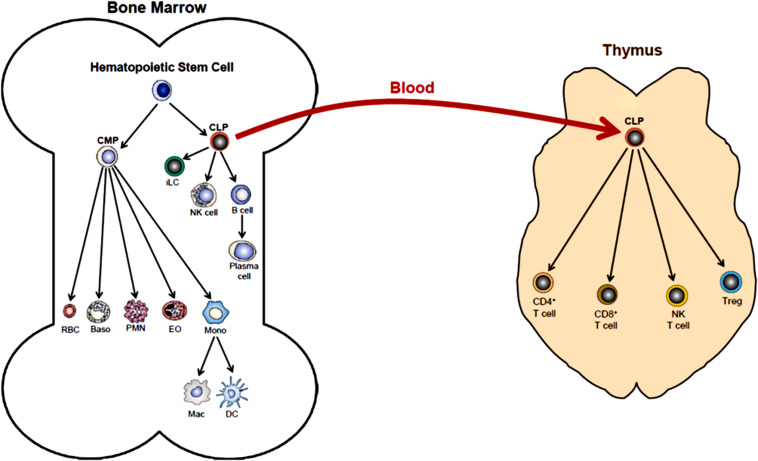
Hematopoiesis. Differentiation of human multi-potent hematopoietic stem cells (HSCs) within the bone marrow (BM) is a complex and highly regulated process that produces most of all cellular elements of blood. The first step in differentiation of HSCs is the generation of lineage-specific progenitor cells called common myeloid progenitor cells (CMPs) and common lymphoid progenitor cells (CLPs). Differentiation of CMPs give rise to erythrocytes (RBC), basophils (Baso), polymorphonuclear neutrophils (PMN, neutrophils), eosinophils (EO) and monocytes (Mono). Monocytes may produce macrophages (Mac) and dendritic cells (DC) within the bone marrow. Monos also enter the blood and traffic to different tissue where they may give rise to Mac and DC. Differentiation of CLPs produce innate lymphoid cells (iLC), natural killer cells (NK cell), B cells and plasma cells. Some BM-residing CLPs will enter the blood and home to the thymus where they will differentiate into at least four additional immune cell populations including CD4+ T cells, CD8+ T cells, NK T cells and regulatory T cells (Treg). (This figure was modified from OpenStax Anatomy and Physiology, May18, 2016, with permission: Download for free at http://cnx.org/contents/14fb4ad7-39a1-4eee-ab6e-3ef2482e3e22@8.25).

The body is continuously exposed to a variety of different bacteria, viruses and fungi which can produce potentially lethal infections. Defense against these unwanted invaders is mediated by a series of synchronized and highly regulated responses that are referred to as innate and adaptive immunity (Figure 1). These two arms of the immune system not only protect against pathogenic microbes, but they are also capable of recognizing and eliminating abnormal cells such as tumor cells. Innate immunity provides for the rapid response to invading pathogens whereas adaptive immunity requires more time to initiate its multifaceted, antigen specific responses. The innate immune system consists of myeloid cells that include polymorphonuclear neutrophils (neutrophils), monocytes, macrophages, eosinophils, basophils and dendritic cells (DCs) as well as innate lymphoid cells (iLCs), natural killer (NK) cells and natural killer T (NKT) cells. The adaptive immune system consists of T and B cells. Most innate immune cells begin their development within the bone marrow (BM) from self-renewing, multi-potent hematopoietic stem cells (HSCs) that will give rise to lineage-specific myeloid progenitor and lymphoid progenitor cells (Figure 2). Common myeloid progenitor cells give rise to all granulocytes (neutrophils, eosinophils, basophils), monocytes, erythrocytes, and platelets. The second major progenitor cell in the BM is the common lymphoid progenitor (CLP) cell that produces NK cells and B cells (Figure 2). Once produced, all myeloid cells, NK cells and B cells are released into the systemic circulation where they continuously patrol the body for evidence of microbial invasion. Circulating monocytes will ultimately leave the circulation and enter different tissues where they undergo maturation resulting in the formation of tissue macrophages (Macs) and DCs. Unlike innate immune cells, T cells undergo a different developmental pathway. Some of the BM-residing CLP cells that give rise to NK, iLC and B cells enter the systemic circulation and traffic to the thymus where they differentiate into MHC class I (MHC I)-restricted CD8^+^ T cells, MHC class II (MHC II)-restricted CD4^+^ T cells and NK T (NKT) cells (Figure 2). Mature CD8^+^ T cells are referred to as cytotoxic T lymphocytes (CTLs) and are very effective at recognizing and killing host cells infected with intracellular pathogens (viruses, bacteria) or tumor cells. The thymus also produces a second lineage of CD4^+^ T cells called thymus-derived regulatory T cells (tTregs) (Wing et al., 2019). These T cells express the transcription factor FOXP3 that is crucial for maintaining tolerance to self or autoantigens in the periphery. Although tTregs represent the majority of Tregs with in lymph nodes, BM and spleen, antigen activation of naïve CD4^+^ T cells residing within the microenvironment of certain tissues such as the small and large bowel will induce the expression of Foxp3 thereby becoming peripheral Tregs (pTregs) (Wing et al., 2019; Campbell and Rudensky, 2020). Naïve CD4^+^ T cells released by thymus will traffic to lymphoid tissue in search of their cognate antigens that are likely derived from invading microbes. Recognition of these antigens bound to MHC II on the surface of antigen presenting cells (e.g., DCs, Macs, B cells) will result in their activation, differentiation and clonal expansion of different subsets of antigen-specific T cells that produce a myriad of cytokines and chemokines. These mediators facilitate the recruitment and activation of other immune cells including macrophages, neutrophils, eosinophils, and basophils to sites of infection and inflammation where they help to destroy pathogenic micro-organisms. This section will discuss SIRT1 in immune cell development and how dysregulation may contribute to autoimmune and chronic inflammatory diseases.

### Hematopoiesis

Numerous studies have suggested that SIRT1 plays an important role in HSC homeostasis, maturation, and lineage determination. Using global (i.e., germline) deletion of SIRT1, Leko et al. (2012) reported that *Sirt1^–/–^* mice contained frequencies and tissue distribution of hematopoietic progenitor populations as well as absolute numbers of differentiated blood cells similar to what was observed in wild type littermate controls. In addition, these investigators found that *Sirt1^–/–^* BM cells were capable of sustained reconstitution when transplanted into lethally irradiated recipients. Researchers concluded that SIRT1 signaling is dispensable in HSC homeostasis in mice. Because neonatal mortality in *Sirt1^–/–^* mice is relatively high with surviving littermates developing severe ADs (Zhang J. et al., 2009), investigators have generated mice with cell-specific deletion of Sirt1 to assess the role of this deacetylase in hematopoiesis. Singh et al. (2013) generated conditional Sirt1-deficient mice by interbreeding Sirt1-E4^floxed/floxed^ mice to mice containing the 4-hydroxy-tamoxifen (4-OHT)–inducible ERT2-Cre transgene or by breeding Sirt1-E4^floxed/floxed^ mice to mice containing the *vav-iCre* transgene which is predominantly expressed in BM progenitor cells. Both *Sirt1-E4^*fl/fl*^/ERT2^*Cre*^* and *Sirt1-E4^*fl/fl*^/vav^*iCre*^* were fed 4-OH for 11 weeks to ablate the *Sirt1* gene. These investigators demonstrated loss of Sirt1 in all tissues (*Sirt1-E4^*fl/fl*^/ERT2^*Cre*^* mice) or in BM-residing progenitor cells (*Sirt1-E4^*fl/fl*^/vav^*iCre*^*) promoted abnormal expansion of HSCs when mice were subjected to hematopoietic stress via administration of cytotoxic or genotoxic drugs (Singh et al., 2013). These atypical responses were associated with a high frequency of genomic mutations, DNA damage and loss of hematopoietic progenitor cells. In another study, Rimmele et al. (2014) reported that the conditional ablation of SIRT1 using a tamoxifen-inducible mouse model, resulted in defective self-renewal properties of HSCs, anemia and lymphocytopenia that was associated with an expansion of myeloid progenitor cells (i.e., granulocyte-monocyte progenitors).

It is well-known that HSCs in the BM reside and interact with a highly specialized microenvironment (called the BM niche) that actively regulates HSC homeostasis. The BM niche contains different stromal and progenitor cells, endothelial cells, extracellular matrix components and a number of secreted factors and surface receptors. Osteoblasts are stromal cells that underly the endosteal bone surface and are thus the major interface between calcified bone and the BM. This stromal niche has been shown to play a crucial role in regulating HSC renewal and expansion (Morrison and Scadden, 2014). Park et al. (2019) recently investigated the role that SIRT1 plays in the osteoblastic niche of BM. These investigators created mice with SIRT1 deficiency in the osteoblastic niche of BM by crossing *Sirt1*^*flox/flox*^ mice with *Ocn*^*Cre*^ mice. Ocn^*Cre*^ mice are used to target osteoblasts in the BM niche. They observed that deletion of SIRT1 in BM osteoblasts did not alter circulating numbers of blood lymphocytes, neutrophils, monocytes, erythrocytes and platelets when compared to controls. In addition, Park et al. (2019) showed that *Sirt1*^floxed/floxed^/*Ocn*^*Cre*^ mice generated similar numbers of CD11b^+^ myeloid cells, T cells and B cells in the BM as did control mice. Furthermore, deletion of SIRT1 in the osteoblastic niche did not alter maturation of HSCs in mice subjected to hemopoietic stress induced by transplantation of HSCs into lethally irradiated or 5-fluorouracil- treated recipients. Park et al. (2019) concluded that in contrast to data reported by Rimmele et al. (2014) who showed that selective deletion of SIRT1 in HSCs caused anemia, lymphocytopenia and increased myeloid cell generation, deletion of SIRT1 in the osteoblastic niche had no effect on HSC maturation or lymphoid and myeloid lineage distribution in the BM.

### Myeloid Cells

Neutrophils, monocytes, Macs, and DCs are the myeloid/innate immune cells that rapidly respond to and destroy invading pathogens. Much of what we have learned about the role of SIRT1 in the regulation of myeloid cell function has come from studies focused on DCs and Macs. Because DCs and Macs are required for robust T cell activation within lymphoid and non-lymphoid tissues, defects in their ability to endocytose (or phagocytose), process and present antigens would have dire consequences for host immunity. Thus, it is important to understand the different molecular and cellular mechanisms involved in DC and Mac function.

Dendritic cells play a crucial protective role following invasion of pathogenic microorganisms by promoting antigen-specific responses by T and B cells as well as by generating a plethora of different chemokines and cytokines that recruit immune cells into the infected tissue where these mediators influence the differentiation of T cells into different effector cell subsets. It is also becoming well-appreciated that SIRT1 plays an important role in immune cell function (Chen et al., 2015). For example, Legutko et al. (2011) demonstrated that pharmacological inhibition of SIRT1 using sirtinol and cambinol suppressed Th2 effector cell responses allergic airway inflammation in a mouse model of asthma. They observed that SIRT1 inhibition interfered with the maturation and migration of lung DCs to the draining lymph nodes where they failed to induce Th2 cell differentiation (Legutko et al., 2011). Because both inhibitors have been shown to inhibit SIRT1 and SIRT2, Legutko et al. (2011) repeated their studies using mice with DC-specific (Yang et al., 2013) deletion of SIRT1 (*Sirt1^*flox/flox*^/CD11c^*Cre*^* mice). They observed reduced maturation and migration of DCs as well as an overall attenuation of Th2-mediated, antigen (OVA)-induced airway inflammation in these mice. Furthermore, these investigators reported that DC differentiation toward a pro-Th2 phenotype required SIRT1-mediated inhibition of peroxisome proliferator-activated receptor-γ (PPARγ). Using the same mice as Legutko and coworkers, Yang et al. (2013) have reported that DCs obtained from *Sirt1^*flox/flox*^/CD11c^*Cre*^* mice exhibited increased production of IL-27 and IFN-β. They reported that co-culturing LPS-activated SIRT1-deficient DCs with CD4^+^ T cells suppressed Th17 differentiation that was reversed by addition of anti-IL-27 and anti-IFN-β antibodies. These data suggested that SIRT1 acts as a negative regulator of IL-27 and IFN-β expression. Consistent with this hypothesis, these investigators demonstrated that DC-associated SIRT1 interacts with and deacetylates the transcription factor IRF-1 resulting in loss of transcriptional activity and suppression of IL-27 expression. Taken together, these data suggest that DC-associated SIRT1 plays an important role regulating Th17 differentiation during inflammation (Yang et al., 2013). Because both IL-27 and IFN-β function to suppress the generation of pro-inflammatory Th17 cells, Yang and coworkers reasoned that *Sirt1^*flox/flox*^/CD11c^*Cre*^* mice may be less susceptible to induction of chronic inflammation. Indeed, they found that loss of SIRT1 function in DCs attenuated the development of chronic neuro-inflammation in a model of human multiple sclerosis called experimental autoimmune encephalomyelitis (EAE) (Yang W. B. et al., 2012). In addition, Liu et al. (2015) reported that DC-specific deletion of SIRT1 via the generation of *Sirt1^*flox/flox*^/CD11c^*Cre*^* mice or SIRT1 inactivation of human DCs resulted in enhanced generation of IL-12 but suppressed production of TGF-β. Molecular studies revealed that DC-associated SIRT1 regulates IL-12 and TGF-β1 expression via HIF1α but not mTOR modulation. These data were consistent with their observation that targeted loss of SIRT1 in DCs induces the formation of Th1 cells while limiting the formation of Tregs (Liu et al., 2015). Similarly, Liu et al., 2015 tested their hypothesis *in vivo* using a well-established mouse model of Th1 cell-mediated inflammatory bowel disease. To do this, they transferred naïve CD4^+^ T cells into *RAG-1^–/–^* mice devoid of DC SIRT1 (*Sirt1^*flox/flox*^/CD11c^*Cre*^/RAG-1^–/–^*) or into DC-replete *RAG-1^–/–^* recipients and monitored the mice for signs of disease. They observed that adoptive transfer of T cells into *Sirt1^*flox/flox*^/CD11c^*Cre*^/RAG-1^–/–^* mice induced a marked acceleration in weight loss and an exacerbation of colonic inflammation when compared to their *RAG-1^–/–^* counterparts at 3 weeks post T cell transfer (Liu et al., 2015). The development of more severe colitis was associated with an increase in IFN-γ producing Th1 cells and reduction in FOXP3^+^ Tregs compared to T cell-engrafted RAG-1^–/–^ mice. Co-culturing of LPS-activated mouse or human DCs with their corresponding CD4^+^ T cells in the presence of the selective SIRT1 inhibitor EX-527 recapitulated much of what was observed using mouse DCs with targeted deletion of SIRT1 including increases in the Th1/Treg ratio, IFN-γ and IL-12 expression as well as reductions in expression of mouse and human TGF-β1 expression (Liu et al., 2015). Taken together, these data demonstrate that DC-associated SIRT1 plays an important role in regulating the balance between Th1 cells and Tregs.

Extravasation of circulating monocytes from the blood and into the tissue initiates their maturation/differentiation into Macs. These phagocytes are well-known to play an important role in the immunopathogenesis of autoimmune and chronic inflammatory diseases. Importantly, Macs have been shown to proliferate in response to inflammatory cytokines and growth factors. Imperatore et al. (2017) recently reported that SIRT1 plays a crucial role in regulating self-renewal of Macs. They have shown that over- expressing SIRT1 in BM-derived Macs during differentiation enhanced their proliferative activity whereas pharmacologic inhibition or SIRT1 gene silencing (via shRNA) or deletion (via CRISPR/Cas9) diminished steady state and cytokine-induced proliferation of alveolar and peritoneal Macs *in vitro* and *in vivo* (Imperatore et al., 2017). Reduction in cell cycle progression and renewal induced by SIRT1 inhibition or gene silencing was associated with inhibition of transcription factors those promote cell cycle progression (e.g., E2F1 and Myc) as well as activation of a transcription factor that is known to induce cell cycle arrest (i.e., FoxO1). Another transcription factor that plays an important role in Mac-mediated immunity is NF-κB. Under steady state, this heterodimeric transcription factor (p50/p65) is localized to the cytoplasm via its binding to the inhibitor protein kB (IkB). Activation of Macs results in the degradation of IkB allowing NF-κB to translocate to the nucleus, where it binds and promotes inflammatory gene expression (Karin and Lin, 2002). It is also well-appreciated that SIRT1 inhibits NF-κB signaling by binding to and deacetylating the p65 subunit (Yeung et al., 2004). Schug et al. (2010) utilized *Sirt1^*flox/flox*^/lysozyme^*Cre*^* to assess how the loss of SIRT1 in myeloid cells affects NF-κB signaling in these immune cells. They found that deletion of SIRT1 in myeloid cells caused hyperacetylation of the p65 subunit in NF-κB resulting in enhanced transcription of inflammatory cytokines including TNF-α and IL-1β in response to TNF-α or LPS stimulation *in vitro or in vivo* (Schug et al., 2010). In addition, these investigators showed that *Sirt1^*flox/flox*^/lysozyme^*Cre*^* mice responded to a high fat diet with the infiltration of large numbers of activated Macs into the liver and adipose tissue that appeared to exacerbate insulin resistance and metabolic syndrome (Schug et al., 2010). Consistent with these observations, Yoshizaki et al. (2010) demonstrated that siRNA-mediated knockdown of SIRT1 in cultured RAW264.7 mouse macrophages enhanced LPS-induced phosphorylation of IkB kinase leading to an increase in IkB degradation, NF-κB activation, and enhanced expression of inflammatory genes. Taken together, these studies suggest that Mac-associated SIRT1 plays a crucial role in modulating NF-κB -mediated inflammation. Another transcription factor that is regulated by SIRT1 is activator protein-1 (AP-1) which is composed of the two submits c-Fos and c-Jun that together regulate inflammatory gene transcription. Zhang et al. (2010) found that SIRT1 directly interacts with the basic leucine zipper domains of c-Fos and c-Jun where it deacetylates c-Jun resulting in suppression of AP-1 transcriptional activity in peritoneal Macs. They also showed that SIRT1-mediated deacetylation of c-Jun reduced the AP-1 mediated expression of the two inflammatory mediators cyclooxygenase-2 and prostaglandin E2 resulting in diminished tumoricidal function (Zhang et al., 2010). Similarly, Yoshizaki et al. (2010) reported that SIRT1 actively suppresses inflammatory gene expression as siRNA knockdown of SIRT1 in cultured Macs enhanced LPS-induced phosphorylation of c-Jun N-terminal kinase (JNK) and c-JUN resulting in increased expression of several inflammatory genes including TNF-α, IL-1β, MMP-9, MCP-1 and IL-6 (Yoshizaki et al., 2010).

### T and B Lymphocytes

**FIGURE 3:**
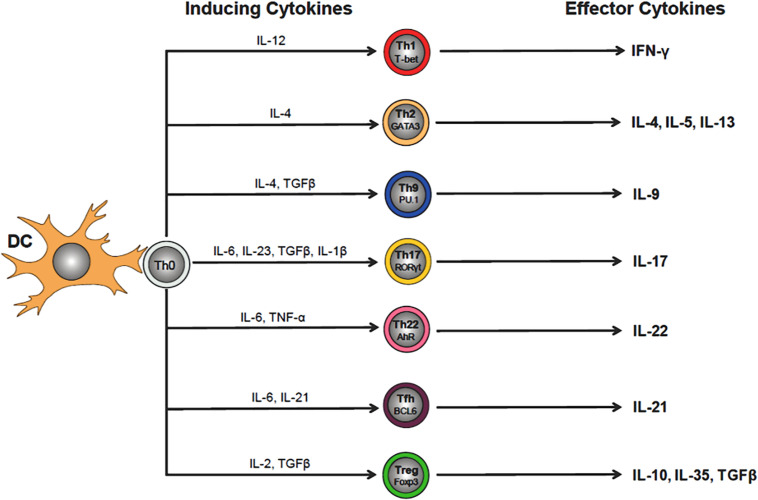
Differentiation of CD4+ T helper (Th) cell subsets. Interaction of naïve (Th0) CD4+ T cells with its cognate antigen presented by dendritic cells (DCs) in the context of MHC II, induces their activation and differentiation into a variety of different Th cell subsets that is dependent upon the cytokine milieu of the microenvironment (e.g., IL-12 for Th1 cells). Th cell-inducing cytokines will upregulate lineage specific transcription factors that regulate and maintain Th cell-specific effector cell functions (e.g., Tbet for Th1 cells). The effector cytokines produced by the different Th subsets (e.g., Th1 cell-generated IFN-γ) act to eliminate the invading microbes that were responsible for the release of the inducing cytokines by DCs during Th0 cell activation.

The adaptive immune system is essential for mounting an effective and well-regulated immune response to invading microorganisms. This highly integrated immune response requires the participation of thymus-derived CD4^+^ and CD8^+^ T cells that is called cell-mediated immunity together with BM-derived B cells and plasma cells which mediate humoral immunity via their production of antibodies (Figure 1). Both cell-mediated and humoral immunity work in close association with the innate immune system to insure protection via long-term memory of infectious agents. As described above, interaction of CD4^+^T cells with their cognate (i.e., microbial) antigen presented by antigen presenting cells in the context of MHC II results in the activation, differentiation and clonal expansion of a variety of different Th cell subsets including T helper 1 (Th1), Th2, Th17, pTreg, Th9 and T follicular (T_FH_) cells (Russ et al., 2013) (Figure 3). The lineage-specific differentiation of the different Th effector cells depends upon the cytokines produced by the antigen presenting cells during their interaction with naive T cells. T_FH_ cells are a specialized subset of thymus-derived CD4^+^ T cells that are produced and reside within lymph nodes, spleen and tonsils where they provide “help” to B cells to produce antibodies with high affinity that neutralize and remove pathogens (Ma et al., 2012). Differentiation of CD4^+^ T cells is a complex process that is mediated by different cytokines, transcription factors and epigenetic alterations (Russ et al., 2013; Ellmeier and Seiser, 2018) (Figure 3). SIRT1 was originally reported to be a negative regulator of T cell function. Zhang J. et al. (2009) reported that CD4^+^ T cells obtained from *Sirt1*^–/–^mice responded to *in vitro* activation with greater proliferation and cytokine production than did wild type T cells. In addition, these investigators showed that *Sirt1*^–/–^ mice developed more severe brain inflammation in the EAE model suggesting loss of tolerance to autoantigens (Zhang J. et al., 2009). However, subsequent studies have demonstrated that targeted deletion of SIRT1 in T cells actually promotes the generation of FOXP3-expressing Tregs with enhanced immunosuppressive activity (Chadha et al., 2019). For example, Beier et al. (2011) found that T cells obtained from mice with a T cell-specific deletion of SIRT1 (i.e., *Sirt1^*flox/flox*^/CD4^*Cre*^* mice), were remarkably similar to wild type mice with respect to T cell numbers as well as their activation, proliferation and cytokine production *in vitro*. In addition, these investigators showed that targeted deletion of SIRT1 in conventional CD4^+^FOXP3^–^ T cells enhanced the expression of FOXP3 in these T cells resulting in the generation of Treg with immunosuppressive activity *in vitro* and *in vivo*. In fact, targeted deletion of SIRT1 in thymic-derived Tregs enhanced significantly their immunosuppressive properties (Beier et al., 2011). In addition, these investigators showed that major MHC mismatched heart allografts survived significantly longer when transplanted into *Sirt1^*flox/flox*^/CD4^*Cre*^* mice or *Sirt1^*flox/flox*^/Foxp3^*Cre*^* mice when compared to allograft transplantation into wild type mice or when transplanted into wild type mice and treated with selective SIRT1 inhibitors (Beier et al., 2011). More recent work by Levine et al. (2016) reported that MHC mismatched renal allografts survived significantly longer with better function when transplanted into *Sirt1^*flox/flox*^/CD4^*Cre*^* vs. wild type mice or when transplanted into wild type recipients and treated with a selective SIRT1 inhibitor. These preclinical studies have prompted discussion to the use of selective targeting of T cell-associated SIRT1 as a possible therapeutic strategy to treat allograft transplantation rejection (Wang et al., 2018). One explanation for the differences in phenotypes and immune responses of T cells derived from *Sirt1*^–/–^ mice vs. T cells obtained from *Sirt1^*flox/flox*^/CD4^*Cre*^* mice is most likely due to alterations in thymic T cell selection in *Sirt1*^–/–^ mice. It is well-known that SIRT1 plays an important role in regulating the expression of the transcription factor, autoimmune regulator (AIRE) that is required for robust thymic T cell selection (Chuprin et al., 2015). Indeed, this may be the reason why *Sirt1^–/–^* mice develop spontaneous and severe ADs that resembles Type 1 diabetes (Sequeira et al., 2008; Zhang J. et al., 2009).

Interaction of naïve CD4^+^ T cells with DCs (or other antigen presenting cells) in the presence of IL-6, IL-23 and TGFβ induces the formation of Th17 cells (Figure 3). Although these IL-17 producing effector cells play a protective role against fungal and bacterial pathogens, Th17 cells have also been implicated in the immunopathogenesis of certain ADs (Omenetti et al., 2019). SIRT1 is highly expressed in Th17 and plays a major role in their generation. It has been well-described that SIRT1 binds to and deacetylates the transcription factor retinoid acid receptor related orphan receptor gamma (RORγt). The deacetylated form of RORγt induces differentiation of Th17 effector cells by activating the IL-17 promoter while repressing the IL-2 promoter (Lim et al., 2015). Lim et al. (2015) demonstrated that targeted deletion of T cell-associated SIRT1 in *Sirt1^flox/flox^CD4^cre^* mice reduced Th17 differentiation by suppressing IL-17 expression while enhancing IL-2 generation. In addition to its ability to enhance the generation of Th17 cells, SIRT1 may also limit Th17 differentiation via its ability to deacetylate signal transducer and activator of transcription (STAT)-3 which is required for RORγt transcription (Limagne et al., 2017). In fact, a few studies have reported that enhancing rather than inhibiting SIRT1 may suppress Th17 development (Gardner et al., 2013, 2015). In order to directly assess the role of SIRT1 in Th17-mediated ADs, Lim et al. (2015) interbred *Sirt1*^*flox/flox*^ mice with RORγt*^*Cre*^* mice producing offspring with selective deletion of Sirt1 in RORγt expressing T cells (i.e., *Sirt1^*flox/flox*^/*RORγt*^Cre^* mice). Using these novel mice or a selective SIRT1 inhibitor (i.e., EX-527), these investigators observed marked suppression of Th17 differentiation, neuronal inflammation and demyelination in the EAE mouse model of multiple sclerosis (Lim et al., 2015). Taken together, these studies suggest that SIRT1 plays a pro-inflammatory role in Th17 effector cells and that selective inhibition of this deacetylase may be useful in treating certain ADs. A third group of Th cells that appear to be regulated by SIRT1 are Th9 effector cells (Figure 3). These IL-9 secreting effector cells have been shown to possess anti-parasitic and antitumor activities; however, other studies have suggested that Th9 cells may be important mediators of allergic diseases (Kaplan et al., 2015). Work by Wang et al. (2016) has demonstrated that SIRT1 serves to negatively regulate the Th9 cell differentiation. They found that targeted deletion of SIRT1 in mouse CD4^+^T cells *(Sirt1^*flox/flox*^CD4^*cre*^*) or SIRT1 silencing in mouse or human T cells (via siRNA inactivation) enhanced Th9 cell differentiation and production of IL-9 (Wang et al., 2016). Conversely, they showed that ectopic expression of SIRT1 reduced IL-9 production and Th9 effector cell numbers. In addition, Wang et al. (2016) found that IL-9 produced by SIRT1 deficient T cells delayed melanoma growth and enhanced development of allergic airway inflammation *in vivo*. In the following sections of this review, we will elaborately discuss T cell mediated SIRT1 roles in multiple ADs (section “The Role of SIRT1 in Autoimmune Disease”).

A second group of T cells that are critically important in cell-mediated immunity against viral infections and tumor cells are the CD8^+^ T cells. Interaction of naïve CD8^+^ T cells with their cognate antigen expressed on the surface of most cells in the context of MHC I, induces antigen-specific activation, differentiation and clonal expansion of cytotoxic lymphocytes (CTLs). Although the role of epigenetic regulation is recognized as an important determinant for CD8^+^ T cell differentiation (Henning et al., 2018), relatively little is known about the role that SIRT1 plays in the generation and activity of CTLs. Kuroda et al. (2011) have shown that suppressing the expression of basic leucine zipper transcription factor, ATF-like (BATF) in CD8^+^ T cells via insertion of GFP cDNA into the *Batf* locus reduced CD8 differentiation *in vivo*. They observed that in the presence of IL-12, co-stimulation of CD8^+^ T cells enhanced expression of BATF, a transcription factor that is required for IL-12 mediated acetylation of histone proteins as well as survival and differentiation of CD8^+^ T cells. In addition, Kuroda et al. (2011) demonstrated that both BATF and c-Jun were responsible for negatively regulating transcription of SIRT1 thereby promoting histone acetylation and expression of the critical transcription factor T-bet. In the absence of BATF, Kuroda et al. (2011) showed that SIRT1 expression was greatly increased in activated CD8^+^ T cells and was associated with deacetylation of T-bet and limited T cell differentiation. Taken together, these data demonstrate that BATF and c-Jun promote CD8^+^ T cell differentiation by negatively regulating SIRT1 expression. Another group of lymphocytes whose function is required for mediating humoral immunity are B cells and their terminally differentiated progeny, plasma cells. These lymphocytes are responsible for generating antibodies that bind, neutralize, and eliminate extracellular microbes and their toxins. Gan et al. (2020) recently reported that SIRT1 plays an important role in regulating antibody maturation in B cells. They found that activation of B cells leads to the down regulation of SIRT1 with concurrent upregulation of activation-induced cytidine deaminase (AICDA), the master regulator of secondary antibody diversification (Gan et al., 2020). Using B cells obtained from mice with B cell-specific ablation of SIRT1 (*Sirt1^*flox/flox*^/Aicda^*Cre*^* mice), Gan et al. (2020) found reduced deacetylation of histone proteins associated with the *AICDA* promoter and non-histone proteins (e.g., Dnmt1 and p65/NF-κB) in activated B cells resulting in increased expression of AICDA and induction of CSR/SHM antibody maturation. Conversely, transgenic overexpression of SIRT1 in B cells was found to reduce expression of AICDA and CSR/SHM confirming its role in silencing AICDA expression. Interestingly, Gan et al. (2020) observed that loss of SIRT1 in activated B cells resulted in the production of several systemic lupus erythematosus (SLE)-associated autoantibodies including antibodies to nuclear antigens, double-stranded DNA, histones, ribonucleoprotein, and RNA IgG. In addition, these investigators reported that B cells obtained from mice that develop spontaneous SLE (e.g., MRL/Faslpr/lpr and BXD2 mice) or from patients with SLE, exhibited increased expression of AICDA that was associated with decreased SIRT1 expression when compared to healthy control mice or humans. Taken together, these data suggest that loss of SIRT1 expression in B cells may promote SLE by driving the generation of SLE-associated autoantibodies.

### The Role of SIRT1 in Infection

Upon invasion of the body by an infectious organism, cells of the immune system undergo proliferation and differentiation, and specify specific lineages that combat the infection by triggering a transition in immune cells from quiescent to an activated state. A key change in this activation involves a metabolic switch within the immune cells that equip them to meet the energetic requirements. For example, myeloid cells undergo a metabolic switch from oxidative phosphorylation during quiescence to glycolysis during activation. This switch to glycolysis is often triggered by Toll-like receptor (TLR) activation which promotes the robust expression and production of inflammatory cytokines. Metabolic reprogramming is important and during immune cell activation can favor specific immune responses. While the shift from oxidative phosphorylation to glycolysis may favor myeloid cell activation, the energy derived may be used to generate more reactive oxygen species in the case of Macs (O’Neill and Pearce, 2016) or synthesize more fatty acids in the case of DCs (Everts et al., 2012). Regardless of the specifics of these metabolic shifts, SIRT1 has been shown to regulate key metabolic pathways including glycolysis, gluconeogenesis, fatty acid synthesis and oxidative phosphorylation. SIRT1 exerts its influence on metabolic pathways by regulating chromatin structure through histone deacetylation as well as through deacetylation of non-histone proteins including transcription factors such as SREBP, a master regulator of fatty acid synthesis (Simmons et al., 2015) or mTOR/HIF1α signaling that regulate genes controlling glycolysis (Wang et al., 2016). NF-κB activity is also regulated by SIRT1 deacetylation, where in Macs SIRT1 deficiency drives an increase in pro-inflammatory cytokines in response to LPS (Schug et al., 2010). Myeloid cells can also be targeted by parasitic infections. One type of parasitic infection spread by insects that can directly impact myeloid cells and modulate metabolic reprogramming involves trypanosomes, which are unicellular protozoa belonging to the *trypanosoma* genus. For example, infection of host cells by *Trypanosoma cruzi* (Tc) can lead to Chagas disease (CD) and a range of mild to severe pathologies. Tc infection can lead to enlargement of the liver, spleen and lymph node and in the case of severe disease can lead to inflammation of the brain and heart and fatal accumulation of fluid around the heart. *In vivo* models of CD show that administration of a SIRT1 activator (SRT1720) suppresses oxidative stress and inflammation associated with chronic Tc infection (Wan et al., 2016, 2019). Wan et al. (2019) demonstrated that SRT1720 had no significant effect on the total population of splenic cells but caused a significant decrease in subpopulations of monocytes and Macs in the spleen of chronically infected mice and a decrease in the CD80^+^/CD64^+^ M1 phenotype Chagas mice. Interestingly, investigators demonstrated that SRT1720 administration had no effect on parasite survival and persistence as measured by parasite DNA in splenic monocytes/macrophages, while there was a significant reduction in the mono/mac ratio in the spleen and heart and an improvement of left ventricular function in Chagas mice. Further investigation of links with SIRT1 and other parasitic infections by other groups found an association with Leishmania, another genus of trypanosomes, which is responsible for the disease leishmaniasis. *Leishmania infantum* (*L. infantum*) which can infect Macs were also shown to trigger metabolic switches in host cells infected with intracellular *L. infantum*. Moreira et al. (2015) demonstrated that infection of Macs with *L. infantum* induces a switch from early glycolytic metabolism to oxidative phosphorylation in a manner that is dependent on SIRT1, liver kinase B1, and AMPK (Roy et al., 2019). Liver kinase B1 plays a key role in HSC maintenance while AMPK critically regulates autophagy in monocytes and Macs based on cellular energy levels. More investigation of other parasites including *Leishmania donovani* (*L. donovani*) further revealed the importance of the competition for metabolic resources between the normal host cell and the parasite-infected host cell. Therefore, the SIRT1 signaling axes linked with AMPK activity has proven to be critical for metabolic programs that influence the pathology associated with parasitic infections (Moreira et al., 2015; Roy et al., 2019).

## The Role of SIRT1 in Autoimmune Disease

Autoimmune diseases are a heterogeneous group of diseases affecting 8–10% of the Western population, characterized by the loss of ability of the immune system to differentiate self from non-self. Nowadays, AD patients are subjected to comorbidities, short life expectancy and progressive disability as the current therapeutic strategies are based on systemic immunosuppression (IS) (Alexander et al., 2019). Even though this is a diverse group of diseases, recent research using genome-wide association studies show that the immune pathogenesis shared by the major ADs is dictated by distinct pathways (Cho and Gregersen, 2011), in which SIRT1 plays an integral role (Table 1).

**TABLE 1:** Summary of immune regulatory pathways and their interaction with SIRT1 in modulating autoimmune diseases (ADs).

Pathway	Gene	SIRT1	References
Lymphocyte activation	Cytotoxic T-lymphocyte antigen 4 (CTLA4)	• In Tregs lymphocytes, depletion of SIRT1-increases mRNA levels of CTLA4.	Beier et al., 2011
	Tumor necrosis factor α-induced protein 3 (TNFAIP3)	• In macrophages, SIRT1 binds to TNFAIP3 loci and participates in its downregulation.	Li et al., 2020
	Tumor Necrosis Factor Receptor Superfamily Member 5, CD40 Antigen (CD40)	• In HUVEC endothelial cells, SIRT1 inhibits TNF-α- induced CD40 expression by deacetylating the RelA/p65 subunit of NF-κB. • In LPS-stimulated renal inner medullary collecting duct (IMCD) cells, SIRT1 overexpression or activation by SRT1720 diminished the expression of CD40, which was reversed by SIRT1 siRNA or inhibitors EX-527 and sirtinol. • In 3T3-L1 adipocytes, SIRT1 regulates TNF-α-induced expression of CD40 via NF-κB pathway.	Lin et al., 2012, 2017; Yang L. et al., 2012
Cytokines and cytokines receptors	Interleukin 23 Receptor (IL23R)	• In T cells, the treatment with a low dose of metformin (SIRT1 agonist) decreases the expression of IL23R.	Limagne et al., 2017
	Interleukin 2 Receptor Subunit Alpha (IL2RA)	• In macrophages, SIRT1 participates in downregulation of IL2RA. • In Treg lymphocytes, the transfection of miR-124a and miR-155 (miRNAs repressing SIRT1) induce the expression of IL2RA.	Heyn et al., 2016; Li et al., 2020
	Interleukin 10 (IL10)	• In microglia (CNS), resveratrol (SIRT1 activator) induces IL10 mRNA expression.	Song et al., 2014
Transcription factors	Signal transducer and activator of transcription 3 (STAT3)	• SIRT1 induces STAT3 deacetylation • SIRT1-null MEF cells display increase of STAT3 levels in mitochondria through constitutive activation of NF-κB	Nie et al., 2009; Bernier et al., 2011

### Role of SIRT1 in Inflammatory Bowel Disease (IBD)

A group of inflammatory diseases that are driven by certain Th effector cells are the inflammatory bowel diseases (IBD; Crohn’s disease ulcerative colitis). These diseases are characterized by chronic and unrelenting inflammation of the small and/or large intestine (Uhlig and Powrie, 2018). There is good evidence suggesting that chronic gut inflammation may arise from a complex interaction among genetics, the immune system and the intestinal microbiota (Uhlig and Powrie, 2018). Akimova et al. (2014) utilized two different models of IBD to ascertain the role of T cell associated SIRT1 in the induction and/or perpetuation of chronic colitis in mice. In their first series of studies using the well-established T cell transfer model, they found that adoptive transfer of syngeneic CD4^+^CD25^–^FOXP3^–^ T cells obtained from wild type C57BL/6 (Bl6) into immunodeficient Bl6 RAG-1^–/–^ recipients induced Th1 effector cell-mediated chronic and unrelenting colitis that was characterized by expansion of disease producing Th1 effector cells that is associated with weight loss, splenomegaly and the infiltration of large numbers of T cells into the colon (Akimova et al., 2014). In contrast, adoptive transfer of Bl6 T cells devoid of SIRT1 (obtained from *Sirt1^*flox/flox*^/CD4^*Cre*^* mice) into Bl6 RAG-1^–/–^ recipients, induced less weight loss and splenomegaly as well as much milder disease when compared to mice that received wild type T cells. The protective effect of eliminating SIRT1 in CD4^+^ T cells corresponded with a 2.8-fold increase in the generation of iTregs when compared to WT T cell engrafted recipients (Akimova et al., 2014). These data suggest that in the absence of SIRT1, differentiation of naïve T cells is skewed toward the generation of iTregs. Using a second model of colitis, Akimova et al. (2014) induced chronic colitis via multiple cycles of drinking water containing the colitogenic polymer dextran sodium sulfate (DSS) that were alternated with drinking water alone. They found that daily administration of the selective SIRT1 inhibitor EX-527 markedly attenuated weight loss and chronic colitis as well as increased the production of iTregs (Akimova et al., 2014). Similarly, Caruso et al. (2014) examined the expression, regulation, and function of SIRT1 in IBD. They found that relative to normal controls, SIRT1 RNA and protein expression was less pronounced in whole biopsies and lamina propria mononuclear cells (LPMCs) of IBD patients. They also reported that SIRT1 expression was downregulated in control LPMC and upregulated in IBD LPMC by neutralizing TNF-α and IL-21 antibodies. Moreover, they found that infliximab-treated IBD patients showed increased SIRT1 expression in mucosal samples and IBD LPMC treated with a specific SIRT1 activator showed reduced NF-κB activation and inhibited inflammatory cytokine synthesis while a SIRT1 inhibitor increased IFN-γ in control LPMC. Overall, assessing patient samples and mouse models of experimental colitis, using a combination of SIRT1 activators and inhibitors, they conclude that SIRT1 is downregulated in IBD patients and colitic mice, and suggest that SIRT1 activation can help attenuate inflammatory signals in the gut (Caruso et al., 2014). Together, these data suggest that T cell associated SIRT1 may represent a potential therapeutic target for treating patients with IBD.

### Role of SIRT1 in Type 1 Diabetes (T1D)

Aside from intestinal/GI-linked autoimmune pathologies, SIRT1 has also been linked with type I diabetes (T1D) and mutations in the SIRT1 gene have been identified in a family with T1D (Biason-Lauber et al., 2013). SIRT1 has been shown to be prominently expressed in beta cells and regulates insulin secretion. In mice, targeted overexpression of SIRT1 in beta cells enhances insulin secretion. In view of this, a mutation in the SIRT1 gene could lead to autoimmune disease. Biason-Lauber and investigators studied a SIRT1 mutation (L107P) that was found in a family harboring this mutation. Four of the five family members developed T1D while 1 in 5 developed colitis. To understand how the mutation might be linked with autoimmune defects, the investigators tested the known functions of SIRT1 and observed whether these functions were altered because of the mutation. Analysis of SIRT1-L107P influence on cytokine production showed increased cytokine-induced nitric oxide synthase expression and TNF-α relative to the wildtype SIRT1 gene. Moreover, in a mouse model of pancreatic insulitis induced by streptozotocin, *Sirt1*^–/–^ mice showed increased islet destruction and hyperglycemia. They also reported that myoblasts from patients harboring this mutation generated insulin resistance in the mice (Biason-Lauber et al., 2013).

### Role of SIRT1 in Rheumatoid Arthritis (RA)

One of the most prevalent ADs in the US is rheumatoid arthritis (RA). This chronic inflammatory disorder is characterized by progressive and unrelenting immune cell-mediated destruction of cartilage and bone in the joints. It has been well-described that myeloid cells such as monocytes and Macs play important roles in the development and perpetuation of chronic joint inflammation (McInnes and Schett, 2011). In an attempt to better understand the role that myeloid cell derived SIRT1 plays in the pathogenesis of experimental arthritis, Hah et al. (2014) used the well-characterized mouse model of arthritis in which serum from arthritic K/BxN mice was injected into control or *Sirt1^*flox/flox*^/lysozyme^*Cre*^* mice. Joint inflammation in recipients is promoted by the presence of autoantibodies directed against the self-antigen, glucose-6-phosphate isomerase that leads to the formation of immune complexes within joints. These immune complexes will activate complement as well as a number of innate immune cells such as Macs, neutrophils and possibly DCs (Christensen et al., 2016). Hah et al. (2014) demonstrated that transfer of serum from arthritic K/BxN donors induced more severe arthritis in *Sirt1^*flox/flox*^/lysozyme^*Cre*^* mice that correlated with significant increases in protein levels of IL-1β and TNF-α as well as increases in TRAP-positive osteoclasts and F4/80+ macrophages in the ankles. BM-derived monocytes and Macs obtained from these mice with selective deletion of SIRT1 exhibited hyperacetylation of p65 and activation of NF-κB that correlated with enhanced polarization, migration and inflammatory cytokine production in these phagocytes when compared to control cells. These data suggest that SIRT1 may act to limit the activation of innate immune cells resulting in less joint inflammation. Although this model induces robust arthritis in 100% of most mouse strains, it is more of an acute form of arthritis that does not involve interactions among T cells, B cells and innate immune cells that are required for the development of chronic disease. Mouse models of chronic arthritis require a priming step in which mice are immunized with self-antigens (e.g., collagen II) to induce T and B cell-dependent production autoantibodies. The second (i.e., effector) phase for the development of chronic arthritis is essentially the same as that involved in the serum transfer model. That is, autoantibodies generated during the priming step enter the synovial space where they bind to collagen II and initiate immune complex deposition and joint inflammation. The priming and effector phases in this model of collagen induced arthritis (CIA), are thought occur in human RA (Christensen et al., 2016). Thus, Woo et al. (2016) undertook a follow up study to assess the role of myeloid cell derived SIRT1 in the CIA model. Interestingly, in contrast to their previous study, Woo et al. (2016) observed less joint inflammation and bone loss in *Sirt1^*flox/flox*^/lysozyme^*Cre*^* mice when compared to their wild type controls. Attenuation of disease correlated with reductions in inflammatory cytokines, MMPs and ROR-γT as well as decreases in the numbers of Th1, Th17 and DCs. Indeed, maturation of DCs obtained from *Sirt1^*flox/flox*^/lysozyme^*Cre*^* mice was impaired as was their ability to induce differentiation and proliferation of disease producing Th1 and Th17 effector cells. SIRT1 has also been shown to be increased in patients with RA (Woo et al., 2016). Another study used resveratrol to assess the role of SIRT1 in CIA. Resveratrol has been shown to be protective against cardiovascular disease and has been connected to increased life spans. Resveratrol affects immune response by inhibiting proliferation of spleen cells, production of TNF-α, IL-12 and T and B cell activity by upregulating CTLA4 and down-regulating CD28 and CD80 (Zou et al., 2013). Zou et al. (2013) reported that SIRT1 inhibition in T cells diminished the resveratrol-induced inhibition of T cell activation and also coincided with changes in c-Jun acetylation and the incidence and severity of collagen-induced arthritis.

### Role od SIRT1 in Acute and Chronic Graft Versus Host Disease (GVHD)

In addition to solid organ transplantation (i.e., host vs. graft reaction), preclinical studies have shown that selective deletion of T cell associated SIRT1 provides protection to mice that receive allogeneic hematopoietic stem cell transplantation (HSCT). This protocol is a potential cure for severe and/or relapsing hematological malignancies, ADs or blood disorders (Niederwieser et al., 2016). Unfortunately, the beneficial effects of HSCT are limited by the development of a multi-organ inflammatory condition called acute graft versus host disease (aGVHD) (Zeiser and Blazar, 2017; Toubai et al., 2018). Typically, inflammatory tissue damage involves the gastrointestinal tract, skin, liver and lungs (Zeiser and Blazar, 2017; Toubai et al., 2018). Though the immuno-pathogenesis of aGVHD has not been completely defined, experimental and clinical studies demonstrate that T cells within the donor BM are the major effector cells responsible for mediating inflammatory tissue injury in the different target tissues (Zeiser and Blazar, 2017). A recent study by Daenthanasanmak et al. (2019) demonstrated that engraftment of allogeneic BM supplemented with allogeneic T cells obtained from mice with targeted deletion of SIRT1 in their T cells produced significantly less aGVHD and chronic GVHD (cGVHD) when compared to wild type recipients that received that same allogeneic BM and T cell suspension. They also observed increased generation of Tregs that were derived from the donor T cells. These induced Tregs exhibited greater FOXP3 stability and immunosuppressive activity *in vitro and in vivo.* In addition, Daenthanasanmak et al. (2019) found that administration of the selective SIRT1 inhibitor EX-527 to mice that received wild type allogeneic BM and T cells significantly increased survival of the recipients that was associated with reduced production of IL-17 and IFN-γ by donor T cells. These results suggest that T cell associated SIRT1 may be a possible target for the treatment of acute and chronic GVHD. Together these findings demonstrate an increasing interest between the crosstalk of endocrine, nervous and immune systems and reveal the interaction between gene expression, cytokine release and hormone action in ADs with SIRT1 (Tanriverdi et al., 2003; Silverman and Sternberg, 2008).

## Prospects for Targeting Sirtuins Pharmacologically

Recognition of the epigenetic defects in tumorigenesis and decades of research led to the clinical use of several FDA-approved therapies that target some of the epigenetic “erasers” (Kelly et al., 2010; Cheng et al., 2019). Several class I/II HDAC inhibitors have either been approved (Suraweera et al., 2018) or are currently advancing in various phases of clinical trials (Ramalingam et al., 2010; O’Connor et al., 2013; Foss et al., 2016; Sborov et al., 2017) for the treatment of multiple types of cancer. However, there are no approved therapies that target SIRT1 by increasing or decreasing its activity, but several studies hint of future promise. For example, SIRT1 may be an important therapeutic target in patients whose complications involve chronic inflammatory responses (Dai et al., 2018; Zhou et al., 2018; Wang Y. et al., 2019). Table 2 lists some of the sirtuin inhibitors potentially effective in diseases with dysfunctional endocrine and immune regulation. One of the promising areas where modulating SIRT1 activity therapeutically may be important involves managing cellular dysfunction associated with protozoa infection involving models of CD where SIRT1 regulates myeloid responses to infection (Wan et al., 2016, 2019). Additionally, other parasites including *Leishmania infantum* and *L. donovani* are capable of subverting key SIRT1 signaling axes linked with AMPK activity. Ultimately, this aberrant engagement of SIRT1 leads to a switch in metabolic programs that favor the pathology associated with infection. Consequently, as we learn more about how SIRT1 mediates metabolic reprogramming of infected cells, this could yield potential new therapeutic targets for treatment of leshmaniasis (Moreira et al., 2015; Roy et al., 2019). Studies have also suggested that targeting SIRT1 may also be effective in decreasing neurodegeneration associated with multiple sclerosis (MS), which is linked with progressive disease despite success in reducing inflammation. Application of SIRT1 activators have been reported to both reduce the degeneration associated with decreased ATP synthesis and increased inflammation in MS models (Fonseca-Kelly et al., 2012; Nimmagadda et al., 2017). While these studies are important, it should be noted that the use of some resveratrol-based inhibitors may elicit effects on uncharacterized targets that are modulated independent of SIRT1. Other inflammation-linked diseases in which modulating SIRT1 activity may show benefit include non-alcoholic fatty liver disease (NAFLD) and diet-induced non-alcoholic steatohepatitis (NASH) (Simmons et al., 2015). SIRT1 has been repeatedly shown to be expressed at high levels in tissues that play a dominant role in the regulation of metabolism and in models of NAFLD, targeting SIRT1 ameliorates inflammation associated with these pathologies (Kume et al., 2010).

**TABLE 2:** List of existing potent SIRT inhibitors and their possible therapeutic implications.

Name	Study objective	Treatment group	Study type	Mechanism of action	References
**Clinical research**					
Nicotinamide	Anticancer Activity of Nicotinamide on Lung Cancer	EGFR Mutated Lung Cancer Terminal Stage Patients	Phase II/Phase III clinical trial (active, not recruiting)	Results not published yet	NCT02416739, https://clinicaltrials.gov
EX-527 (Selisistat)	SIRT1 Antagonist Therapy Before Embryo Transfer to Improve Endometrial Receptivity and Life Pregnancy Rates	Endometriosis, Uterine Diseases, Endometrial Diseases, Infertility, Unexplained Infertility; Female Non-implantation	Double blind, placebo-controlled Phase II clinical trial	Protective SIRT1 inhibitor functions of EX-527 in Huntington’s disease treatment (Süssmuth et al., 2015)	NCT04184323, https://clinicaltrials.gov
**Preclinical research**					
Nicotinamide	Anticancer effects of SIRT1 inhibitor	Human leukemia and prostate cancer cells	*In vitro*	(i) Blocked cancer cell proliferation and promoted apoptosis via p53 dependent caspase-3 and miR-34a induction (ii) Inhibited cancer cell growth and viability through SIRT1 mediated inhibition of Foxo1 acetylation	Jung-Hynes et al., 2009; Audrito et al., 2011
AK-7	Neuroprotective action via SIRT2 inhibition	Huntington’s disease naïve neuronal cell model	*In vitro*	Brain permeability with limited metabolic stability via induced SREBP-2 cytosolic retention and downregulated transcription of cholesterol biosynthesizing enzymes	Taylor et al., 2011
EX-527	Antinociceptive and anticancer effects of SIRT1 inhibitor	BALB/c Mice injected with bone cancer cells; Human leukemia cells; PABPN1 transgenic nematodes; Primary human mammary epithelial cells	*In vitro*, *In vivo*	(i) induced cellular apoptosis in cancers (ii) protected against muscular dystrophy (iii) Increased P53 acetylation in breast epithelial cells after DNA damage	Solomon et al., 2006; Pasco et al., 2010; Cea et al., 2011; Lux et al., 2019
AC-93253	Anticancer effects via SIRT1-3 inhibition	Prostate DU145, Pancreas MiaPaCa, Lung A549 and NCI-H460 cancer cells	*In vitro*	Cytotoxic effects against a panel of cancer cell lines	Zhang Y. et al., 2009
Inauhzin	Anticancer effects of SIRT1 inhibitor	Human lung cancer, H460 and colon cancer, HCT116 cells; Wildtype and lung/colon tumor bearing SCID mice	*In vitro;* *In vivo*	Reactivated p53 via SIRT1 inhibition thus: (i) Repressed cancer cell proliferation, (ii) Stimulated cancer cell senescence and apoptosis without genotoxicity, and (iii) Repressed the xenograft tumor growth	Zhang et al., 2012
Sirtinol	Anticancer effects of SIRT1/2 inhibitor	Human Breast cancer MCF7, Lung cancer H1299, Prostate cancer PC3 and Du145 cell lines	*In vitro*	(i) Induced senescence-like growth arrest with impaired activation of RAS-MAPK pathway (ii) P53 dependent cell apoptosis (iii) Increased sensitivity to chemotherapeutic agents along with inhibited cell proliferation	Ota et al., 2006; Kojima et al., 2008; Jin et al., 2010; Wang et al., 2012
Salermide	Anticancer effects of SIRT1/2 inhibitor	Human cancer cell lines: Leukemia (MOLT4 and KG1A); Breast cancer (MCF7 and MDA-MB-231); Colon cancer (SW480); and non-small-cell lung cancer lines	*In vitro*	Induces apoptosis, growth arrest and reduces cancer cell proliferation in a panel of cancer cell lines via p53-independent and dependent inhibition of SIRT1/2; have potent cytotoxic and anti-proliferative effects against cancer	Lara et al., 2009; Peck et al., 2010; Liu et al., 2012; Rotili et al., 2012a
Cambinol	Anticancer, inflammatory and immune response effects of SIRT1/2 inhibitor	Human lymphoma and breast cancer cell line; Mouse Burkitt lymphoma model; Bone marrow cells from BALB/c mice housed in pathogen free conditions	*In vitro;* *In vivo*	Promotes cell cycle arrest via hyperacetylation of tubulin, p53, Foxo3a, and KU70; reduced xenograft tumor growth in a mouse Burkitt lymphoma model; reduced neuroblastoma formation in N-Myc transgenic mice; and Repressed aromatase transcription via ERα deacetylation in breast cancer Decreased proinflammatory cytokines expression and macrophage response upon microbial stimulation (TLR) *in vivo*	Heltweg et al., 2006; Marshall et al., 2011; Holloway et al., 2013; Lugrin et al., 2013
Splitomicin and its analogues	Anticancer effects of SIRT1/2 inhibitor	Human colon (DLD-1), cervical (HeLa), breast (MCF7) cancer, and colorectal, glioblastoma cancer stem cells	*In vitro*	Weak or anti-proliferative effects against a panel of human cancer cell lines and cancer stem cells	Neugebauer et al., 2008; Rotili et al., 2010, 2012b
Tenovin-1/-6	Anticancer effects of SIRT1/2 inhibitor	Human breast (MCF7, MDA-MB-231), gastric cancer (MKN-45, NUGC-4, STKM-2, SNU-1), lymphoma (BL2 Burkitt’s), and melanoma (ARN8) cell lines; ARN8 mouse xenograft model	*In vitro;* *In vivo*	Cytotoxic to a panel of cancer cell lines (breast, gastric, lymphoma, melanoma); delayed xenograft tumor growth and disease progression partly via p53 activation and death receptor-5 (DR-5) upregulation	Lain et al., 2008; Hirai et al., 2014; Jin et al., 2015

Apart from the inhibitors listed in Table 2, natural SIRT1 inhibitors are currently under investigation due to their anticancer roles. For instance, Subramaniyan et al. (2017) reported Wnt-mediated inhibition of SIRT1 signaling in SW480 colorectal cancer cells by sodium salt ‘Burtin’ extracted from *Butea monosperma* flowers. Similarly, betulinic acid derivative (B10) increased FOXO3a transcription and reduced SIRT1 expression leading to induced apoptosis *in vitro* and reduced *in vivo* tumor growth and hence B10 is a novel therapeutic candidate for glioma treatment (Huo et al., 2017). It is intriguing that the SIRT1 inhibitor, nicotinamide, is the amide derivative of vitamin B3. This begs the question as to what role does diet or the supplementation of specific vitamins have on disease progression and the regulation of protein/histone acetylation? Along these lines, the cancer-preventative soy peptide, lunasin, is also known to regulate the dynamics of acetylation-deacetylation (de Lumen, 2005). Studies have demonstrated that consumption of soy milk more than once a day is associated with a 70% reduction in prostate cancer risk compared with no soy milk intake (Hebert et al., 1998). Although we are still early in our exploration of the impact of acetylation on protein function, there are several landmarks indicating that the journey is still worth the effort. Taken together, given the potential therapeutic implication of sirtuin inhibitors in cancer and other immune modulatory diseases, good selectivity and optimization practices should be employed for future clinical and preclinical studies.

Finally, examples are increasingly emerging that hint the promise of targeting SIRT1 and its downstream effectors therapeutically that may involve an intersection of endocrinology and immunology. For example, the accumulation of excess lipids and cholesterol esters in arterial walls is characteristic of atherosclerosis, and macrophage-derived foam cells play a critical role in this process. Macrophage-derived foam cells localize to blood vessel walls wherein fat accumulation is enriched, and within this vascular microenvironment, they uptake low-density lipoproteins, thereby promoting atherosclerosis. Retinoid signaling regulates cholesterol deposition and removal in macrophages, in part, by regulating the steroidogenic acute regulatory (StAR) protein (Manna et al., 2016). StAR activity and other pathways linked with retinoid and hormone signaling have been shown to regulate this process. For example, we demonstrated that the activation of cAMP/PKA signaling promotes retinoid-induced macrophage cholesterol efflux and increased LXR activity, a process that may reduce the formation of atherosclerotic lesions. Moreover, we found that treatment of mouse macrophages with retinoids increased cholesterol efflux to apolipoprotein AI (Apo-A1) and elevated StAR promoter activity while macrophages overexpressing hormone-sensitive lipase increased the hydrolysis of cholesterol esters (Manna et al., 2015a). Not only can retinoids modulate StAR activity in macrophages, but they also regulate StAR at multiple levels across a wide spectrum of cell types (Manna et al., 2015b). StAR is well recognized for its ability to mediate intra-mitochondrial transport of cholesterol in target tissues, however, only recently has it been shown to be a target of SIRT1 as well as other lysine deacetylases (Manna et al., 2019). While much remains to be discovered about how endocrine linked signaling modulates key regulators of immune responses, it is clear that the intersection of these fields of study will likely find SIRT1 at the crossroads.

## Concluding Remarks

From these examples and others not discussed at length here (Hu et al., 2014; Dai et al., 2018; Zhou et al., 2018; Wang S. H. et al., 2019), it is apparent that epigenetic drugs that regulate acetylation/deacetylation will likely prove beneficial in the treatment of several diseases. Combination therapies involving deacetylase inhibitors and immunotherapy may provide yet another means for treating numerous disorders. Finally, these agents might also be effective for chemopreventive approaches for individuals who have not yet acquired neoplastic lesions or advanced stage disorders. Likewise, our understanding of the role that SIRT1 plays in cellular transformation and tumorigenesis is still murky; however, there is enough evidence that warrants further investigation of the link. Moreover, it is becoming increasingly appreciated that SIRT1 plays an important role in innate and adaptive immunity as well as in the pathogenesis of certain autoimmune or chronic inflammatory diseases. Studies presented in this review reveal that the role of this histone deacetylase is context specific, as SIRT1 acts as a positive or negative regulator of gene expression depending upon the specific immune cell and its environment. For example, T cell associated SIRT1 appears to play an important role in promoting the generation of Th1, Th9 and Th17 effector cells as well as inducing certain autoimmune or chronic inflammatory diseases. In contrast, B cell associated SIRT1, in the steady state, appears to act as a negative regulator for the production and maturation of protective antibodies. Many of the studies described in this review discuss the therapeutic potential of cell specific SIRT1 targeting in the treatment of different autoimmune and chronic inflammatory disease. Additionally, evidence demonstrates that chronic inflammation and autoimmunity are associated with the development of malignancy (Franks and Slansky, 2012; Yu et al., 2016) and patients with a primary malignancy may develop autoimmune-like disease (Valencia et al., 2019). These relationships imply a need for deeper understanding of the role of up- or down-regulation of SIRT1 and surveillance of patients on immunomodulatory therapies for potential secondary disease processes.

## Author Contributions

All the authors were involved in manuscript writing and revision. FR and KP finalized the draft. FR submitted the manuscript with consent from all authors.

## Conflict of Interest

The authors declare that the research was conducted in the absence of any commercial or financial relationships that could be construed as a potential conflict of interest.
